# Pesticides and Parkinson’s Disease: The Legacy of Contaminated Well Water

**DOI:** 10.1289/ehp.117-a553a

**Published:** 2009-12

**Authors:** Tina Adler

**Affiliations:** **Tina Adler** first wrote for *EHP* about the Clinton–Gore environmental agenda in 1993. She is a member of the National Association of Science Writers and the American Society of Journalists and Authors

Epidemiologic studies since the early 1990s have suggested that exposure to various classes of pesticides increases the risk of developing Parkinson’s disease (PD). Animal studies have backed up that link, revealing how pesticides may target the dopaminergic system, which is damaged in PD. New data from the Parkinson’s Environment and Genes Study show that residents of California’s Central Valley who over many years drank well water that was probably highly contaminated with certain pesticides were more likely to have PD or PD symptoms than residents who didn’t drink contaminated well water **[*EHP*** **117:1912–1918; Gatto et al.]**.

Private wells are at risk of pesticide contamination because pesticides can drift several hundred meters from application sites and travel through the soil. Moreover, many of the private wells studied were less than 15–20 yd deep, lessening the likelihood that pesticides will have degraded by the time they reach the water supply. Unlike municipal water supplies, private well water is not required to be monitored for contamination.

The researchers analyzed long-term data on pesticide application rates near the homes of the study participants, who included 368 people clinically confirmed to have possible or probable PD and 341 controls. The authors had access to 26 years’ worth of data collected under California’s mandated pesticide use reports program on the commercial application of pesticides, including where the pesticides were applied, on what date, and in what quantities. They studied 26 pesticides that were potential groundwater contaminants or that had been previously linked to PD.

The researchers combined those data with land-use maps, which the California Department of Water Resources updates every 7–10 years, to pinpoint more precisely where pesticides had been applied. Using geographic information system software, they were able to merge historical data on home addresses, land use, and pesticide applications. The result was a prediction of amounts of pesticides applied per acre per year within 500 m of the study participants’ homes.

People with PD were more likely to get their water from private wells and to have drunk well water longer than controls. Whereas people with exposure to ambient pesticides—essentially, proximity to sites where pesticides were applied—were 15–57% more likely to be classified as having PD than people without ambient exposure, those with combined ambient exposure and exposure via well water potentially contaminated with methomyl, chlorpyrifos, or propargite were 67%, 87%, or 92% more likely to be cases. The odds of PD also increased as the number of different pesticides that potentially contaminated a subject’s drinking water increased.

Unlike previous research on the link between PD and pesticide exposures, this study used a semiquantitative approach to estimating pesticide exposure and did not rely on study subjects’ recall. Also, all PD cases were clinically confirmed by a movement disorder specialist. The results therefore considerably strengthen the evidence that exposure to pesticides in well water may contribute to PD.

